# Validation of the GLIM Malnutrition Criteria in Patients With Gastric Cancer at the Time of Diagnosis

**DOI:** 10.1155/grp/6636000

**Published:** 2026-08-16

**Authors:** Paola Sánchez-Corrales, Yazmín Hernández-Murillo, Luis Enrique Orozco-Rivera, Vanessa Fuchs-Tarlovsky, Gabriela Gutiérrez-Salmeán

**Affiliations:** ^1^ Centro de Investigación en Ciencias de la Salud (CICSA), Facultad de Ciencias de la Salud, Universidad Anáhuac México, Huixquilucan, Estado de México, Mexico, anahuac.mx; ^2^ Servicio Soporte Nutricional, Hospital Dr. Rafael Ángel Calderón Guardia. Caja Costarricense de Seguro Social, San José, Costa Rica; ^3^ Centro Avanzado de Investigación Multidisciplinaria en Nutrición y Metabolismo, Hospital General de México “Dr. Eduardo Liceaga”, CDMX, Mexico, hgm.salud.gob.mx; ^4^ Escuela Militar de Graduados de Sanidad, Secretaría de la Defensa Nacional, CDMX, Mexico

**Keywords:** gastric cancer, Global Leadership Initiative on Malnutrition (GLIM), malnutrition, Patient-Generated Subjective Global Assessment (PG-SGA), validation criteria

## Abstract

Gastric cancer is a major global public health challenge, with high incidence and mortality rates, particularly in Costa Rica. Like most oncological diseases, it significantly wastes nutritional status, often leading to malnutrition, which in turn worsens prognosis and survival. Early identification of malnutrition is therefore critical. The Global Leadership Initiative on Malnutrition (GLIM) has proposed standardized diagnostic criteria to systematically diagnose malnutrition across diverse clinical settings. However, validation has not been performed versus reference methods in many conditions, such as gastric cancer, although it is essential before widespread adoption. This study is aimed at validating the GLIM criteria for the timely detection of malnutrition in patients newly diagnosed with gastric cancer. We conducted a cross‐sectional analysis in which GLIM‐based assessments were compared against the Patient‐Generated Subjective Global Assessment (PG‐SGA) as the gold standard. Diagnostic performance was evaluated through sensitivity, specificity, and positive and negative predictive values; agreement was assessed with kappa statistics. Survival probability was further examined using Kaplan–Meier curves to estimate the prognostic relevance of nutritional status. A total of 72 patients were included. GLIM criteria demonstrated a sensitivity of 81.9% and specificity of 81.8% compared with PG‐SGA, with moderate agreement (kappa = 0.48). Survival analysis revealed that both GLIM and PG‐SGA consistently identified better outcomes among patients with preserved nutritional status. In conclusion, the GLIM criteria represent a valid and practical tool for the early detection of malnutrition in gastric cancer patients at diagnosis, supporting their integration into routine clinical practice to improve patient management and survival outcomes.

## 1. Background

Gastric cancer is a public health problem that, in 2020 according to the Global Cancer Observatory (GLOBOCAN), represented 5.6% of all reported cancer cases, and an increase of up to 68% is expected by 2025 [1]. In Costa Rica, the prevalence in that same report was a total of 952 cases, which represents 7.2% of cases of cancer, a percentage higher than the global avaerage [1].

Cancer‐associated malnutrition profoundly amends body composition through complex metabolic and inflammatory mechanisms. Tumor‐derived and host‐mediated secretion of proinflammatory cytokines promote metabolic pathways enhancing catabolism thus yielding involuntary weight loss, depletion of skeletal muscle and adipose tissue. Such alterations are strongly associated with increased mortality, higher complication rates, and impaired functional status in cancer patients, underscoring the clinical relevance of early recognition and intervention [2–4].

Considering the above‐described different malnutrition diagnostic methods have been studied, including the Subjective Global Assessment generated by the patient (PG‐SGA), which is considered the reference standard in cancer patients [5].

Historically, the diagnosis of malnutrition in clinical practice lacked a standardized and universally accepted framework, resulting in heterogeneous methodologies and fragmented data that limited comparability across studies and populations. This absence of a homogeneous diagnostic approach hindered the generation of concordant evidence and restricted the development of unified therapeutic strategies. To address this gap, an international consortium of experts proposed the Global Leadership Initiative on Malnutrition (GLIM) criteria, establishing a structured diagnostic scheme that integrates phenotypic and etiologic indicators to harmonize malnutrition assessment worldwide [6]. The GLIM framework has since been validated in multiple populations, including hospitalized medical patients, surgical cohorts, older adults, and oncology patients, consistently demonstrating strong associations with adverse outcomes such as increased complications, reduced treatment tolerance, and higher mortality [7–10]. However, despite their growing global adoption, these criteria have not yet been specifically validated in Costa Rican patients with gastric cancer at the time of diagnosis—a population with one of the highest incidences of this malignancy worldwide. Such validation is essential to ensure accurate detection, facilitate early nutritional intervention, and generate evidence that is both locally relevant and internationally comparable, ultimately contributing to improved patient care and survival outcomes [6].

Thus, the objective of this study was to validate the GLIM criteria as a tool for the detection of malnutrition in patients with gastric cancer at the time of diagnosis and as a secondary objective the comparison of said criteria against the PG‐SGA in a third‐level hospital in Costa Rica.

## 2. Materials and Methods

### 2.1. Study Design

A cross‐sectional study, from June 2021 to July 2022, was carried out, in which the GLIM and PG‐SGA tools were applied at the time of gastric cancer diagnosis. Eligibility criteria were as follows: patients over 18 years of age, patients being referred to the Nutritional Support Service at Hospital Dr. Rafael Ángel Calderón Guardia by medical or surgical oncology services, and patients with diagnosis of gastric cancer (confirmed with positive biopsy). Noninclusion criteria were as follows: patients being pregnant, patients with presence of pacemaker, patients with previous diagnoses of cancer, patients with previous cerebrovascular events, and patients with nephropathy and/or liver disease. Sampling was carried out through consecutive cases. Data was collected by two clinical nutrition specialists and a trained general practitioner; in all cases, the same interviewer applied both diagnostic tools (GLIM and PG‐SGA).

### 2.2. Data Collection

Anthropometric measurements were taken in all cases. Weight was obtained with a SECA Model 813 scale and height with a SECA model 213 stadiometer, using the techniques described by Carmenate et al. [11]

Information was obtained in all cases from primary sources (i.e., the patient himself and his companions), except for the laboratory values that were obtained from the digital registries of the Costa Rican Social Security Fund hospitals.

• Subjective Global Assessment Generated by the patient (PG‐SGA): The seven items that correspond to the subjective global assessment generated by the patient were completed, the patients themselves completed the part that corresponds to them according to the test and the rest was completed by the corresponding research physician. Subsequently, each patient was assigned to the corresponding categories: A, without malnutrition; B, moderate malnutrition; or C, severe malnutrition [5].
• GLIM criteria: Both the phenotypic and etiological criteria of the GLIM were completed [6]. To categorize the patient as malnourished, at least one phenotypic criterion must have been met, which defined the severity of malnutrition, and one etiological criterion. For the criterion of reduced muscle mass, multifrequency bioimpedance was used, with InBody S10, with the patient seated. All patients were asked not to perform physical exercise 24 h in advance and fasted for at least 4 h prior to performing the bioimpedance to determine the fat‐free mass index and the phase angle [6]. Cutoff points of the described parameters were considered from those proposed by the European Society of Clinical Nutrition and Metabolism (ESPEN) for fat‐free mass (fat‐free mass/height in meters squared): men 17 kg/m^2^ and women 15 kg/m^2^ [12, 13].

### 2.3. Ethical Considerations

The present study was approved by both Research (CICSA202101) and Bioethical (CEC‐062021) Institutional Committees. An informed consent was signed by each participant.

### 2.4. Statistical Analysis

Qualitative variables are presented as percentages and rates, and for quantitative variables, the mean and standard deviations were calculated. For the validation of tests, sensitivity, specificity, and positive and negative predictive values were calculated. The kappa coefficient was also calculated to evaluate the concordance between the applied instruments. Furthermore, chi‐squared and odds ratio (OR) were assessed, using logistic regression to evaluate the relationship between PG‐SGA and GLIM.

Finally, survival was evaluated with Kaplan–Meier curves and hazard ratio, using a Cox regression to establish the risk of death according to the degrees of malnutrition. Statistical significance was considered when *p* < 0.05.

## 3. Results

A total of 78 patients with a diagnosis of gastric cancer were referred to the Nutritional Support Service between the months of July 2021 and July 2022. Of these, six participants were excluded due to the following reasons: absence of a malignant tumor on postsurgical biopsy, a final diagnosis of lymphoma, a diagnosis of chronic liver disease, a diagnosis of chronic kidney disease, and a refusal to answer the required questionnaires in two cases. Of the participants, 72.2% were men. Mean age was 65 years (minimum 31, maximum 90) and 16.7% were under 45 years old. Findings in accordance with both GLIM and PG‐SGA (without malnutrition, with moderate malnutrition and with severe malnutrition) are detailed in Table 1. PG‐SGA yielded malnutrition in 84.8%, whereas the GLIM criteria present this condition in 73.6%; moderate and severe grading were higher when considering the PG‐SGA.

**Table 1:** Malnutrition prevalence in gastric cancer patients.

Diagnosis	GLIM	PG‐SGA
Without malnutrition	26.4%	15.2%
Moderate malnutrition	23.6%	30.6%
Severe malnutrition	50%	54.2%

Abbreviations: GLIM, Global Leadership Initiative on Malnutrition; PG‐SGA, Patient‐Generated Subjective Global Assessment.

Table 2 presents validation of the GLIM criteria with the reference standard (PG‐SGA). They presented adequate specificity and sensitivity according to the parameters stipulated by the GLIM group. Regarding the agreement between these tests, it is concluded that in both tests it is moderate according to the kappa coefficients obtained. Regarding the OR, there is an association between the comparisons with OR values > 2 (GLIM vs. PG − SGA = 20.45), which allows validating the GLIM criteria not only against the standard of reference.

**Table 2:** Validity for malnutrition diagnosis for GLIM versus PG‐SGA criteria.

Test statistic	GLIM recommended interpretation	GLIM vs. PG‐SGA values
Sensitivity	> 80%	81.9%
Specificity	> 80%	81.8%
Positive predictive value	NA	96.1%
Negative predictive value	NA	45%
Kappa coefficient	0.8	0.78
*X* ^2^	*p* < 0.05 if sample < 200	*p* < 0.05
Odds ratio	≥ 2	20.45

Abbreviations: GLIM, Global Leadership Initiative on Malnutrition; PG‐SGA, Patient‐Generated Subjective Global Assessment.

**Figure 1:**
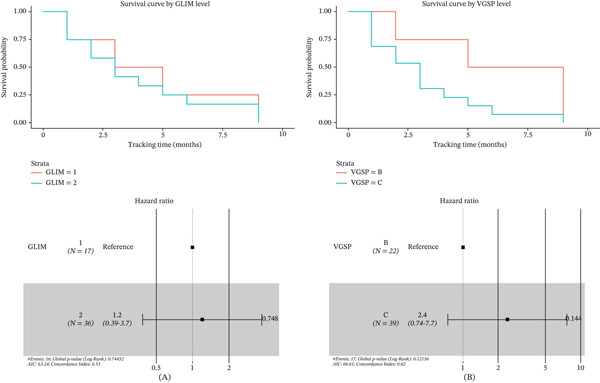
Mortality according to stage of malnutrition on (A) GLIM and (B) PG‐SGA score.

Finally, within the study, the relationship between mortality and the degree of malnutrition obtained by the application of the scales was analyzed, the results are observed in Figure 1, as well as the ORs that establish the relationship of mortality risk according to the stage of malnutrition. According to stage of malnutrition. This figure shows that patients with severe malnutrition have a higher risk of death than those with severe malnutrition, both when the GLIM and PG‐GSA scales are applied.

## 4. Discussion

Malnutrition in gastric cancer arises from complex pathophysiological mechanisms that extend beyond reduced food intake due to tumor‐related anorexia. These patients also present early satiety due to the presence of the neoplasm per se; dyspepsia and acid‐reflux are also common because the tumoral mass increases intragastric pressure thus preventing lower esophageal sphincter from closing. However, systemic inflammation and metabolic alterations play a central role. Proinflammatory cytokines such as interleukins (e.g., IL‐6) and tumor necrosis factor (TNF‐*α*) induce hypercatabolism, proteolysis in both circulating proteins and those from skeletal muscle, and lipolysis, leading to wasting and further cancer cachexia [14, 15].These mechanisms explain why malnutrition is highly prevalent in gastric cancer and why standardized diagnostic tools are urgently needed to ensure timely detection and intervention.

In regards of epidemiology, in this study, we found that the average age of presentation of gastric cancer (65 years old) coincides with the published literature which states that it is more frequent between 50 and 70 years [13–16]. The distribution by sex showed a higher frequency in men, as reported in previous local studies and in GLOBOCAN for Costa Rica, which indicate that the incidence is 8.4% in men and 6.1% in women [1].

Considering malnutrition frequency, our study demonstrated a high prevalence of malnutrition among patients with gastric cancer. Using the PG‐SGA, 69.8% of participants were classified as malnourished, with 23.6% presenting moderate and 50% severe malnutrition. For its side, the GLIM criteria identified a slightly higher prevalence, with 73.6% of patients affected, of whom 30.6% had moderate and 54.2% severe malnutrition. These findings are consistent with international reports, where malnutrition in gastrointestinal cancers frequently ranges between 50% and 80%, worsening in advanced stages due to tumor‐related anorexia, obstruction, systemic inflammation, and treatment‐related toxicities. Importantly and as mentioned before, Costa Rica stands out as a country with one of the highest incidences of gastric cancer worldwide, estimated at 20–25 cases per 100,000 annually, which is significantly higher than neighboring countries such as Mexico (6–8/100,000) and Brazil (7–9/100,000) and comparable to high‐incidence regions like Japan and Korea (30–40/100,000). In these populations, GLIM‐defined malnutrition has been reported in over 70% of advanced gastric cancer patients, with strong associations to poor survival and increased complications. European (from Spain and Italy) studies have also shown GLIM prevalence around 60%–65% in hospitalized gastrointestinal cancer patients, reinforcing the global relevance of these findings [6, 8–10, 17]. The convergence of data across regions underscores the urgent need for systematic worldwide application of GLIM criteria, which would allow for concordant and comparable evidence, strengthen clinical guidelines, and support the development of targeted nutritional interventions. In high‐incidence countries such as Costa Rica, standardized malnutrition diagnosis is particularly critical to integrate nutrition into cancer care pathways and improve patient outcomes: patients with greater malnutrition have higher mortality, as will be specified later [18].

In terms of our primary objective, our findings demonstrate that the GLIM criteria provide good sensitivity and specificity when compared with the PG‐SGA, supporting their validity as a malnutrition diagnostic test in this oncological population. The original consensus report by the working group established the framework and emphasized the need for validation across settings [19], later providing the methodological guidance for validation, which was used in [20]. These guidelines recommend sensitivity and specificity values > 80% for criterion validity, a significant (*p* < 0.05) chi‐square test for construct validity, OR of at least 2 for predictive validity, and at least moderate agreement; all of these were met by the results presented here. With this, our findings contribute to the call of action by the same GLIM group on studies that stated the validation of their criteria; however, they do not clearly state how validation was conducted nor describe methodologically sound. This is extremely important because a global effort is being made to obtain a unified scale and to improve the diagnosis of malnutrition in patients from different populations.

Finally, regarding mortality, according to PG‐SGA, no patients with assessments in A (without malnutrition) died whereas patients classified as C (severe malnutrition) died 2.4 times more than those classified as B (moderate malnutrition). In the GLIM case, only one patient classified as 0 died and those classified as 2 (severe malnutrition) died 1.2 times more than those classified as 1 (moderate malnutrition). This is consistent with what has been described in the literature that directly correlates the severity of malnutrition with a lower survival rate in patients. For example, a study reported that malnutrition and cancer cachexia significantly increase mortality risk across oncological populations, underscoring the need for early nutritional intervention [14]. In gastrointestinal cancers, it has been demonstrated that patients identified as malnourished by GLIM had poorer tolerance to chemotherapy and shorter overall survival [10]. Surgical patients with GLIM‐defined malnutrition experienced increased postoperative mortality and morbidity [9]. These findings emphasize the urgent need for prompt diagnosis of malnutrition using standardized tools such as PG‐SGA and GLIM, followed by optimized nutrition therapy to improve prognosis and potentially reduce mortality. Yet, systematic application of these criteria in clinical practice could therefore play a decisive role in enhancing survival outcomes in gastric cancer and other malignancies.

## 5. Conclusions

This observational study concludes that the GLIM criteria are a valid tool for the diagnosis of malnutrition in patients with gastric cancer at the time of diagnosis, complementing evidence from other populations. Together with mechanistic insights into cancer‐associated malnutrition, these findings underscore the necessity of global implementation of GLIM to harmonize diagnosis, enhance comparability of research, and ultimately improve patient care through timely nutritional support.

Moreover, consistent use of GLIM criteria allows researchers to explore the impact of nutritional status on treatment outcomes, paving the way for targeted nutritional therapies and improved survival.

## Author Contributions

Concept and design of study: P.S‐C., V.F‐T., and G.G‐S. Data acquisition and analysis: P.S‐C., Y.H‐M., and L.E.O‐R. Statistical analysis: P.S‐C. and L.E.O‐R. Wrote the manuscript: P.S‐C., Y.H‐M., and L.E.O‐R. Coordinated the analysis and reviewed the manuscript: V.F‐T. and G.G‐S. V.F‐T. and G.G‐S. are cosenior of this work.

## Funding

This project was financed by the Costa Rican Social Security Fund.

## Disclosure

This project was financed by the Costa Rican Social Security Fund. The funder had no further inquiry in the design, the data, etc. The study was carried out in a center that serves only the enrolled population, therefore a sample from all areas of the country is not included.

## Conflicts of Interest

The authors declare no conflicts of interest.

## Data Availability

The data that support the findings of this study are available from the corresponding author upon reasonable request.
